# Crucial workload variables in female-male elite Brazilian Beach Handball: An exploratory factor analysis

**DOI:** 10.5114/biolsport.2023.114285

**Published:** 2022-06-01

**Authors:** Carlos D. Gómez-Carmona, Daniel Rojas-Valverde, Markel Rico-González, Vinicius C. De Oliveira, Luis Lemos, Clarice Martins, Fabio Y. Nakamura, José Pino-Ortega

**Affiliations:** 1 Grupo de Optimización del Entrenamiento y Rendimiento Deportivo (GOERD). Facultad Ciencias del Deporte, Universidad de Extremadura, Cáceres, Spain; 2 Centro de Investigación y Diagnóstico en Salud y Deporte (CIDISAD), Escuela Ciencias del Movimiento Humano y Calidad de Vida, Universidad Nacional, Heredia, Costa Rica; 3 Clínica de Lesiones Deportivas (Rehab&readapt), Escuela Ciencias del Movimiento Humano y Calidad de Vida (CIEMHCAVI), Universidad Nacional, Heredia, Costa Rica; 4 Department of Didactics of Musical, Plastic and Corporal Expression, University of the Basque Country, UPVEHU. 48940 Leioa, Spain; 5 Physical Education Course Coordinator, International School Paraíba, João Pessoa, Brazil; 6 Research Center in Sports Sciences Health Sciences and Human Development, (CIDESD), Portugal; University of Maia, ISMAI, Portugal; 7 Departamento de Actividad Física y Deporte. Campus de Excelencia Internacional “Mare Nostrum”. Facultad de Ciencias del Deporte. Universidad de Murcia, San Javier, Murcia, Spain

**Keywords:** PCA analysis, Inertial measurement units, Physical performance, Activity patterns, Locomotion

## Abstract

This study aimed to identify the most important variables of male and female beach handball workload demands and compare them by sex. A total of 92 elite Brazilian beach handball players (54 male: age 22.1 ± 2.6 years, height 1.8 ± 0.5 m, weight 77.6 ± 13.4 kg; and 38 female: age 24.4 ± 5.5 years, height 1.7 ± 0.5 m, weight 67.5 ± 6.5 kg) were analyzed in 24 official matches during a four-day congested tournament. From 250 variables measured by the inertial measurement unit, fourteen were extracted for analysis using Principal Component Analysis as selection criteria. Five Principal Components (PC) were extracted that explained 81.2–82.8% of total variance (overview of workload demands during beach handball). Specifically, 36.2–39.3% was explained by PC1 (Distance_Expl_, Distance, Distance_4–7 km/h_, and Acc), 15–18% by PC2 (Acc_Max_, Acc_3–4 m/s_, Dec_4–3 m/s_), 10.7–12.9% by PC3 (Jumps_Avg_ Take-Off, Jumps_Avg_ Landing and PL_RT_), 8–9.4% by PC4 (Distance_> 18.1 km/h_, Speed_Max_), and 6.7–7.7% by PC5 (HR_Avg_ and Step Balance). Sex-related differences were found in the PC distribution of variables, as well as in selected variables (HR_Avg_, Dec_4–3 m/s_, Acc_3–4 m/s_, Jumps_Avg_ Take-Off, Jumps_Avg_ Landing, Acc_Max_, Distance, Distance_4–7 km/h_, Acc, Speed_Max_) with higher values in male players (p < .05). In conclusion, the sex-related PC distribution and workload demands in beach handball should consider for training design and injury prevention programs.

## INTRODUCTION

Beach handball is a recently-created sport, which was developed from handball, and has become a popular discipline with the support from International to Regional Handball Federations [1]. The match consists of two sets of 10-minute where the draw results is resolved with a shoot-out to determine the winning team. Beach handball is practiced in sand surface with specific rules in order to be more spectacular and safer than indoor handball [2]. During the game, players have a specialized role (attack or defense), substitutions are made throughout the lateral band continuously during the game, and a double score for goals scored with 360º throwing, during flights, or by an attacking player dressed as a goalkeeper (specialist) are allowed [1]. These special characteristics do beach handball very attractive, unpredictable, and spectacular sport due to the multiple offensive technical possibilities [3].

The beach handball game model is cyclic, and composed by two alternative phases: (a) ball possession, where the attacking system is structured with numerical superiority due to the substitution of defensive players by offensive ones, and the substitution of the goalkeeper (gk) by the specialist (4 vs 3 + gk); and (b) turnovers, where the defensive system is structured with numerical inferiority, due to the substitution of offensive players by defensive players, and the specialist by the goalkeeper (3 + gk vs 4) [4]. In beach handball, the numerical superiority of the attacking team facilitates successive counterat-tacks, requiring a physical demand, characterized by fast-paced actions in which the two teams pass a ball with the aim of scoring goals [1]. During the game, a combination of high-intensity efforts (e.g. accelerations with short recoveries) are distributed along the match duration [5]. As a result, players are exposed to intermittent demands of high- and low intensities, where appropriate levels of speed, sprint ability, strength, and power are required [1, 6]. Therefore, knowing the physical and physiological requirements is necessary for the adaptation of training sessions to competitive demands [5].

Numerous studies have been carried out to analyze workload demands in indoor handball through the use of inertial and tracking devices [7–9]. However, to date, beach handball is a relatively new sport and there is a lack of information related to its physical demands [2]. Two previous published studies [1, 6] have showed that players covered a total distance of between 1,000 and 1,200 meters. Most of the displacements are performed at low-intensity (0.5–13 km/h), and with low-intensity impacts (5-6G), demanding a moderate physiological response (71–80% of maximal heart rate [HR_MAX_]), though internal and external workload demands depends on different contextual factors, such as competitive level, age or sex of the participants.

Indeed, sex-related differences were found in handball and beach handball with greater distance covered by female players, and higher impacts, accelerations, and high-intensity displacements in male players [1, 8]. Specifically in beach handball, female players covered higher total distance (*p* = 0.049, *d* = 0.79) and distance walking (*p* < 0.001, *d* = 2.04) in the first half, whereas in the second half females covered higher distance standing (*p* = 0.008, *d* = 1.05), obtaining a higher average speed than males (*p* < 0.001, *d* = 2.28) during the match. The number of accelerations distributed over different intensity categories (i.e. low-, moderate-, and high-intensity) was 43.2 ± 11.6, 9.4 ± 4.9, 0.8 ± 0.9 actions for male players, and 40.3 ± 12.7, 4.3 ± 3.0, 0.1 ± 0.3 actions for female ones, which is equivalent to one body acceleration every 23 s and 27 s, respectively [1].

Regarding the internal load variables, male and female players achieved a maximum heart rate (HR_MAX_) of 173 ± 13, and 177 ± 13 bpm, maintained an average HR (HR_AVG_) of 137 ± 12 and 138 ± 18 bpm, and spend a 20.3% and 29.2% of the total time in the anaerobic zone (81–90% HR_MAX_), respectively [1]. In the same way, Gutiérrez-Vargas et al. [6] reported significant differences between male and female players in total distance (*p* < 0.01), average speed (*p* < 0.01), maximum speed (*p* = 0.022), total impacts (*p* < 0.01), body weight change (%) (*p* = 0.038), sweat rate (*p* < 0.01), and fluid intake (ml) (*p* < 0.01). In addition, a significant decrease was found in the maximum speed (*p* = 0.05) and body load (*p* = 0.026) in the second period in both sexes [6]. In this sense, the systematical register and analysis of internal and external workload can provide valuable information to characterize the competition demands and design training session adapted to them [10, 11].

For this systematical register, workload monitoring has become a crucial factor for optimizing players’ performance in sport [12]. Since analyzing the specific requirements of each sport modality depends on the different contextual factors that could influence them, different non-invasive tools have been developed to quantify workload demands during the competition [13]. The workload quantification can be performed for internal [14] and external load through time-motion analysis in indoor [15, 16] and outdoor conditions [17, 18], tactical analysis [13], and neuromuscular load through accelerometry [19] in an objective and accurate way. These sensors allow recording of up to 400 variables per match [20, 21]. However, in a practical setting, fast evaluation of training/competition loads is necessary to assess performance and inform exercise prescription [22], and not all variables are decisive [23]. Therefore, variables that are not relevant or are redundant should be excluded from the assessment to provide more meaningful information to the coaches and athletes.

To address this point, different statistical methods have been applied and principal component analysis (PCA) has been shown to be a suitable approach [24, 25]. This method identifies the most relevant information to explain a phenomenon and excludes the components which are not essential to explain performance. The identification of the variables that explains the internal and external workload demands in beach handball could help team staff to reduce the big data provided by the EPTS, as well as a better understanding on physical and physiological requirements during the official games [21, 26]. Therefore, the purposes of the present study are to: (a) identify the variables that explain the variance of internal and external workload of elite-level male and female beach handball players, and (b) analyze the sex-related differences of the registered workload during an official tournament.

## MATERIALS AND METHODS

### Study Design

A cross-sectional design with natural groups was implemented to analyze locomotion demands of 6 male and 4 female elite Brazilian beach handball clubs during a 4-day congested fixture tournament using an electronic performance and tracking system (EPTS). A total of 24 matches were played in two days (13 male and 11 female matches), with a total of 152 records obtained from players. All matches were played under official rules and judged by federated referees, taking place in the same venue (Praia do Cabo Branco, João Pessoa, Brazil) in a two-court setting. The environmental conditions were substantially similar during the 4-day tournament, according to the Weather Forecasting and Climate Studies Center, from the Brazilian Government. Temperature ranged between 27.8 and 30.4ºC, the air humidity between 64 and 69% and the wind velocity between 2.57 and 3.08 m/s. The sand was uniformized with a squeegee before each game, to minimize possible sand depth differences. A Principal Component Analysis was used to extract the most representative variables to compare the external workload by sex.

### Participants

Of a total of 92 players, 54 were male (age 22.1 ± 2.6 years, height 1.8 m ± 0.5 m, weight 77.6 ± 13.4 kg) and 38 females (age 24.4 ± 5.5 years, height 1.7 m ± 0.5 m, weight 67.5 ± 6.5 kg). They were selected from top elite Brazilian beach handball teams. The study was conducted according to the Declaration of Helsinki (World Medical Association, 2013) guidelines. All players were informed of the benefits and possible adverse consequences of participation in the study, and the protocol was approved by the Institutional Review Board of the University Federal of Paraiba (Register code: 02896918.1 .0000.5176).

### Instruments and procedures

Time-motion variables were assessed using EPTS (WIMUPRO^TM^, RealTrack Systems, Almeria, Spain) with a Ultra-Wide Band (UWB) radiofrequency tracking system. Participants were equipped with the EPTS 15-min prior to warm-up and asked to wear it during the matches at the height of the 2^nd^ to 4^th^ thoracic vertebrae using a special vest.

IMUs were calibrated following recent research criteria [27]. All data were measured using a sampling frequency of 18 Hz for positioning and 100 Hz for accelerometry-based variables. The accuracy and reliability of the system have previously been reported in different conditions [28].

**FIG. 1:**
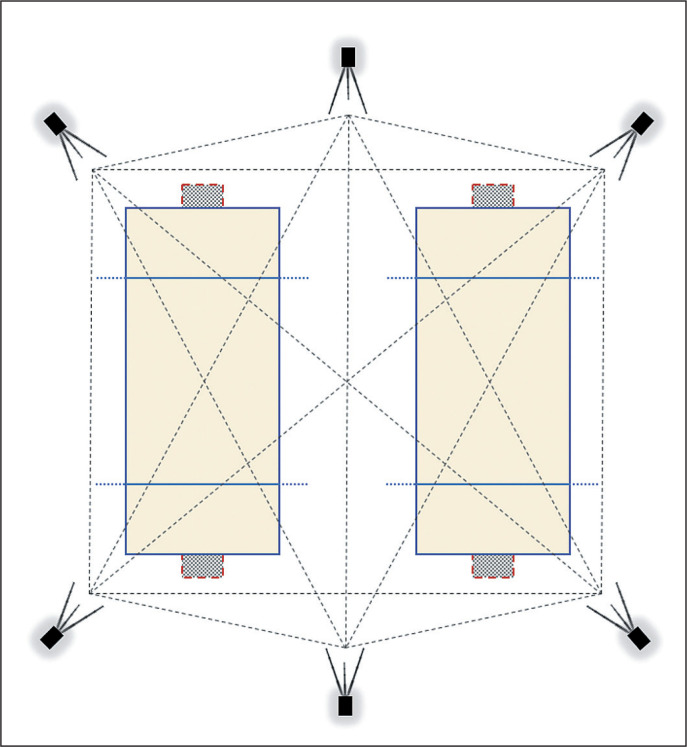
Ultra-Wide Band setting in a two-court beach handball scenario.

As all matches were performed in the same venue, a six antennae UWB system setting was used to cover two side to side courts as shown in figure 1. Antennae were positioned 3.5-m from the floor and metallic material was avoided around the system. The antennae were fixed 4.5–5.5 m from the perimeter of the court in order to form a hexagon shape for better emission and reception of the signal, as shown in figure 1.

All the data obtained from the matches were downloaded and exported immediately after each match finished using proprietary software (SPRO, RealTrack Systems, Almeria, Spain). The criterion to include players in the statistical analysis was participation in > 60% (> 12 min) of total playing time. All break pauses were considered in the analysis (e.g., ball out, fouls, free-throws, and others) in order to explore natural match behavior in an ecological manner, as adopted in other team sport studies [25]. Considering beach handball substitution particularities [8, 9], the software was programmed to automatically identify who was playing and which player was “on the bench”. The software allows the court lines to be pre-set and delimited as well as identification of who is playing and who is resting outside the court (substitutes). In addition, goalkeepers were excluded from the study due to workload demands and technical-tactical actions differ to court players [29, 30].

Due to these particularities in substitution and changes in the position between players during the game, only relative variables expressed relative to time (e.g., relative distance, accelerations per min) and maximum values of a variable were included in the analysis. Considering these potential individual and peer differences by period, positioning and other situational variables there were no discrimination in the inclusion of one-person multiple data from different matches. The limits stablished by Pueo et al. [1] for distance (0–0.4; 0.5–4; 4.1–7; 7.1–13; 13.1–18; > 18.1 km/h), acceleration and deceleration (1–2, 2–3, > 3 m/s^2^), impacts (5–6, 6–6.5, 6.5–7, 7–8, 8–10, > 10 g) and heart rate (< 60%, 61–70%, 71–80%, 81–90%, 91–95%, > 95% HR_MAX_) were utilized. In addition, the acceleration and deceleration zones 3–4, 4–5, 5–6, 6–7, 7–8, 8–9, 9–10 and > 10 m/s^2^ were included in the analysis. After PCA analysis, the fourteen variables extracted were: explosive distance as the distance covered at over 16 km/h (Distance_Expl_, m/min), total distance covered per min (Distance, m/min), distance covered at 4.1–7 km/h (Distance_4.1–7 km/h_, m/min), distance covered over 18.1 km/h (Distance_> 18.1 km/h_, m/min), total accelerations per min (Acc, n/min), maximum acceleration (Acc_Max_, m/s), accelerations made between 3 and 4 m/s per min (Acc_3–4 m/s_, n/min), decelerations made between 4 and 3 m/s per min (Dec_4–3 m/s_, n/min), average g-force in the take-off (Jumps_Avg_ Take-Off, g), average g-force when landing (Jumps_Avg_ Landing, g), maximum speed (Speed_Max_, km/h), average heart rate (HR_Avg_, bpm), step balance percentage that determines the difference between left and right steps (Step Balance, %), and RealTrack player load per min that represented the accelerometry workload in the three planes of movement and was calculated according to Reche-Soto et al. (PL_RT_, a.u./min) [31].

### Statistical analysis

All the variables were reported using the mean and standard deviation. The procedure followed before and after the Principal Component Analysis (PCA) was developed according to previous similar papers in sport [23, 25]. Prior to each PCA analysis (three PCA: general, male, and female), from a total of 250 variables, only maximum and relative variables (n = 129) were selected for correlation matrix exploration, and the variables with r < 0.7 extracted for PCA [32]. Variables were scaled and centered (Z-Score) and PCAs were suitable considering Kaiser-Meyer-Olkin values (KMO = 0.73–0.803) and when Barlett’s Sphericity test was significant (p < 0.01) [33]. After PCA, eigenvalues greater than 1 were included for extraction of the respective principal component (PC) [33]. An orthogonal rotation using the VariMax method was used for identification of respective loadings in each PC; only loadings greater than 0.6 were retained for interpretation and the highest loading was reported when a cross loading was identified between PCs.

An independent *t*-test was used to compare the locomotion performance by sex. Cohen’s *d* was used to report the magnitude of the differences, qualified as follows: *d* < 0.2 as trivial, *d* = 0.2 as small, *d* = 0.5 as moderate, and *d* > 0.8 as large effect size [34]. Alpha was prior set as *p* < 0.05. Data analysis was performed using the Statistical Package for the Social Sciences (SPSS, IBM, SPSS Statistics, v.22.0 Chicago, IL, USA) and graphs were made using Prism software (GraphPad Software, San Diego, CA).

## RESULTS

After general PCA, five different principal components (PC1, PC2, PC3, PC4, and PC5) were extracted (see table 1), which explained 81.2% of the total data set variance. According to respective loadings, the variables were grouped as follows: PC1 explained 39.3% of variance (Distance_Expl_, Distance, Distance_4–7 km/h_, and Acc), PC2 15% (Acc_Max_, Acc_3–4 m/s_, Dec_4–3 m/s_), PC3 12.2% (Jumps_Avg_ Take-Off, Jumps_Avg_ Landing, and PL_RT_), PC4 8% (Distance_> 18.1 km/h_ and Speed-_Max_), and PC5 6.7% (HR_Avg_ and Step Balance).

**TABLE 1:** Results of principal components analysis (orthogonal rotation) with % variance explained by sex

Variable	Men						Women						General					
	M ± SD (LL; UL)	Loading					M ± SD (LL; UL)	Loading					M ± SD (LL; UL)	Loading				
		PC1	PC2	PC3	PC4	PC5		PC1	PC2	PC3	PC4	PC5		PC1	PC2	PC3	PC4	PC5
Eigenvalue		5.8	2.4	1.6	1.4	1.1		5.4	2.7	1.9	1.3	1		5.9	2.2	1.8	1.2	1
%Variance		38.4	16.3	10.7	9.4	7.7		36.2	18	12.9	9.1	6.7		39.3	15	12.2	8	6.7
%Cumulative variance		38.4	54.7	65.4	74.8	82.4		36.2	54.1	67	76.1	82.8		39.3	54.3	66.5	74.6	81.2

Distance_Exp_i (m/min)	4.4 ± 1.9 (0.1; 9.1)	0.62					2.9 ± 1.5 (0.1; 6.1)	0.72					3.6 ± 1.9 (0.1; 9.1)	0.72				

Distance (m/min)	37.7 ± 12.5 (4.1; 57.2)	0.91					28.3 ± 12.6 (4.8; 52.5)	0.92					32.7 ± 13.3 (4.1; 57.2)	0.91				

Distance_4–7 km/h_ (m/min)	11.3 ± 4.1 (0.5; 17.7)	0.88					8.2 ± 4 (0.8; 19.2)	0.87					9.7 ± 4.4 (0.5; 19.2)	0.88				

Distance_18—50 km/h_ (m/min)	0.1 ± 0.5 (0; 3.6)				0.94		0.2 ± 0.2 (0; 1.1)				0.95		0.2 ± 0.4 (0; 3.6)				0.89	

Acc (n/min)	15.3 ± 3.6 (5; 23)	0.72					13.4 ± 4.6 (2.6; 21.1)	0.79					14.3 ± 4.2 (2.6; 23)	0.8				

Acc_Max_ (m/s)	3.1 ± 0.5 (1.5; 4.2)		0.86				2.8 ± 0.5 (1.4; 3.8)		0.79				3 ± 0.6 (1.4; 4.2)		0.8			

Acc_3–4 m/s_ (n/min)	0.2 ± 0.3 (0; 1)		0.93				0.1 ± 0.1 (0; 0.5)		0.93				0.1 ± 0.2 (0; 1)		0.93			

Dec_4–3 m/s_ (n/min)	0.1 ± 0.2 (0; 1.2)		0.87				0.1 ± 0.1 (0; 0.5)		0.93				0.1 ± 0.2 (0; 1.2)		0.89			

Speed_Max_ (km/h)	20.7 ± 8.1 (7.6; 39.1)				0.9		15.8 ± 2.8 (9.6; 28.8)				0.81		18.4 ± 6.6 (7.6; 39.1)				0.78	

HR_Avg_ (bpm)	162.2 ± 23.4 (93; 193)					−0.74	150.1 ± 24.9 (71; 195)	0.79					155.8 ± 24.9 (71; 195)	0.75				

Step Balance (%)	0 ± 0 (−0.2; 0.1)					0.8	0 ± 0 (−0.1; 0.1)					0.97	0 ± 0.1 (−0.2; 0.1)					0.96

Jumps_Avg_ Take-Off (g)	2.8 ± 2.2 (0.8; 13.3)			0.94			1.9 ± 1.7 (0.5; 12.3)			0.89			2.3 ± 2 (0.5; 13.3)			0.92		

Jumps_Avg_ Landing (g)	5.4 ± 2.3 (3; 13.2)			0.8			4.5 ± 1.6 (3.1; 12.2)		0.81				4.9 ± 2 (3; 13.2)			0.8		

PL_RT_ (a.u./min)	1.6 ± 5.1 (0.1; 29.8)			0.93			0.6 ± 1.5 (0.1; 12.7)			0.96			3.7 ± 1.1 (0.1; 29.8)			0.91		

**Note.** PC: Principal component; M: mean; SD: standard deviation; LL: lower limit; UL: upper limit; DistanceExpl: distance covered at over 16 km/h; Acc: Accelerations; Dec: Decelerations; HR: heart rate; PL: player load.

In men, Distance_Expl_, Distance, Distance_4–7 km/h_, and Acc as PC1 explained 38.4% of total variance, Acc_Max_, Acc_3–4 m/s_, Dec_4–3 m/s_ as PC2 explained 16.3%, Jumps_Avg_ Take-Off, Jumps_Avg_ Landing and PL_RT_ as PC3 explained 10.7%, Distance_> 18.1 km/h_ and Speed_Max_ as PC4 explained 9.4%, and HR_Avg_ and step balance as PC5 explained 7.7%. Slightly different distributions of variable loadings were found in women after running PCA, where PC1 explained 36.2% but HR_Avg_ was grouped in this first component, PC2 explained 18% but Jumps_Avg_ Landing was part of this second component, PC3 explained 12.9%, PC4 explained 9.1%, and PC5 with only one variable explained 6.7% of total data set variance.

**FIG. 2:**
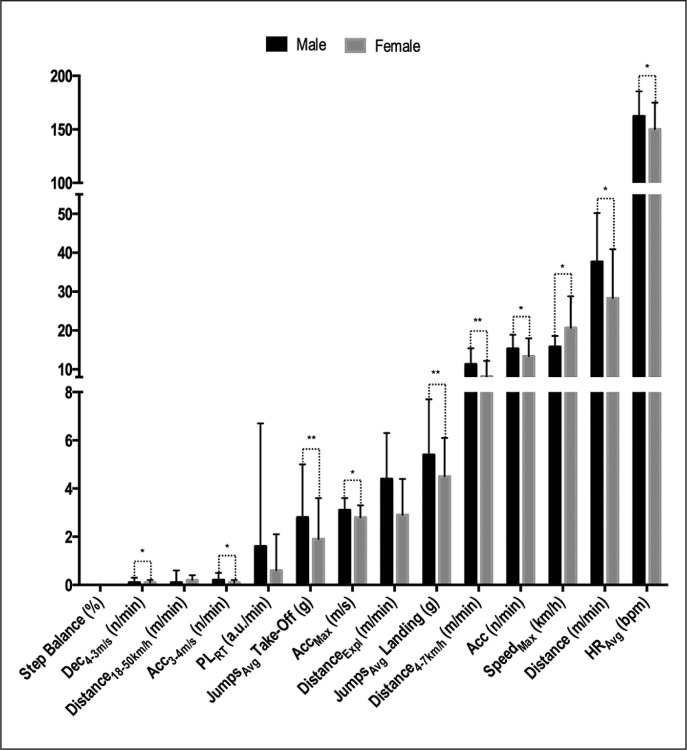
Sex comparison of extracted external and internal workload variables. Significant differences: **p < 0.05, *p < 0.01.

There were significant sex-related differences in HR_Avg_ (*t* = 2.62, *p* < 0.01, *d* = 0.26 [small]), Dec_4–3 m/s_ (*t* = 2.31, *p* = 0.02, *d* = 0.24 [small]), Acc_3–4 m/s_ (*t* = 3.62, *p* < 0.01, *d* = 0.38 [small]), Jumps_Avg_ Take-Off (*t* = 2.38, *p* = 0.02, *d* = 0.24 [small]), Jumps_Avg_ Landing (*t* = 2.32, *p* < 0.02, *d* = 0.24 [small]), Acc_Max_ (*t* = 3.2, *p* < 0.01, *d* = 0.33 [small]), Distance (*t* = 4.13, *p* < 0.01, *d* = 0.43 [small]), Distance_4–7 km/h_ (*t* = 4.29, *p* < 0.01, *d* = 0.45 [small]), Acc (*t* = 2.63, *p* < 0.01, *d* = 0.27 [small]) and Speed_Max_ (*t* = -4.35, *p* < 0.01, *d* = -0.45 [small]); but no significant differences were found in step balance (*t* = 0.62, *p* = 0.54, *d* = 0.06 [trivial]), Distance_> 18.1 km/h_ (*t* = -0.51, *p* = 0.61, *d* = 0.06 [trivial]), PL_RT_ (*t* = 1.55, *p* = 0.12, *d* = 0.16 [trivial]) or Distance_Expl_ (*t* = 0.34, *p* = 0.73, *d* = 0.04 [trivial]) (see figure 2.).

## DISCUSSION

The analysis of specific workloads during competition should be considered in the training planning and recovery protocols to maintain the best physical condition of athletes [12]. Thanks to microtechnology, a large amount of data is obtained, however, in each sport modality it is necessary to select the variables that explain most of the behavior [22]. Through this process, the final report of the evaluation can be concise and rapidly available to team staff to make opportune decisions [21]. Therefore, this study aimed to identify the most important variables of male and female beach handball workload demands and to compare them between sexes. The main results showed that 81.2% of the total data set was explained by five principal components and that sex influenced the workload demands during official matches at elite level.

For the whole sample, five principal components explained 81.2% of the total variance observed. The first principal component is related to volume of demands, both in displacements (Distance, Distance_Expl_, Distance_4–7 km/h_), changes of speed (Acc), and physiological demands (HR_Avg_). The beach handball characteristics impose higher energetic and neuromuscular demands at the same running speeds compared to firm surfaces [35, 36]. Although there are many fewer body contacts (hits and pushes) compared to court handball, the players are required to walk/run on the unstable sand surface, and this fact may increase their physical demands.

Different contextual factors could influence the internal workload demands of the beach handball players. One of them is the level of players because Pueo et al. [1] found lower values in men and women players (137 ± 12 and 138 ± 18 bpm) in comparison with the results of the present study (162.2 ± 24.4 and 150.1 ± 24.9 bpm). Other factors previously identified that could influence the internal workload demands are the type of sand and heat stress. Less compact sand and higher values of temperature and humidity modify the heart rate requirements, requiring higher levels [37, 38]. To avoid the between-match effects, the environmental factors were substantially similar during the 4-days tournament (methods section for more details) and the sand was uniformized with an squeegee before each game. Therefore, it could evidence that the level of the players and environmental factors directly influences the internal load demands during competition. It is important to address the requirements of beach handball players in specific conditions, being reference values for future championships with the same conditions.

The second principal component highlighted in the current study is related to high-intensity changes of speed (Acc_Max_, Acc_3–4 m/s_, Dec_4–3 m/s_). In beach handball, the distances traveled while sprinting are shorter than on the court, since there is up to 15-m to run between the lines demarcating the goal areas. The high-intensity changes in speed (15%) are related to the game dynamics designed to perform counterattacks continuously, as well as having great influence on the decision-making ability of the athlete [30]. Thus, as a consequence, the acceleration speed on a sand surface needs to be addressed in ecological situations as it may be considered an important determinant of player´s capacity.

The third component observed is related to jump capacity and neuromuscular load (Jumps_Avg_ Take-Off, Jumps_Avg_ Landing and PL_RT_). In the same way as changes in speed, jumps represent high importance in the locomotion demands in the game (12.2%) due to the double score for goals scored in flight or 360º [3]. Considering that landing following jumps produce high loads for athletes, being associated with injuries and overuse conditions [39], and landing was highlighted as a PC in the current study, future studies should further explore this variable and its impact on athletes’ performance.

The fourth principal component, which explains 8% of the variance, is related to maximum intensity displacements (Speed_Max_ and Distance_> 18.1 km/h_). Due to the smaller beach handball playing space, the 15-meter “valid” court depth does not allow players to develop maximum speed [40]. This component has a low explanation in beach handball athlete performance compared with other sports with large dimensions, such as basketball (22.3%) [25] or soccer (36.2%) [23]. Therefore, it is important to focus training on the physical qualities of beach handball players related to competition demands.

Finally, the fifth component is related to step balance. This principal component analysis could be explained by the unstable surface where matches are played, and the large number of accelerations and decelerations performed in competition matches that explain 6% of beach handball behavior.

When analyzing male and female principal components, no differences were found between sexes. This fact could indicate that the game dynamics and key factors that explained the beach handball behavior are similar in both sexes. Instead, men performed greater demands in all external and internal workload demands, except in Speed_Max_. Considering that male athletes are more effective when throwing, it is assumed that female matches are characterized by a greater number of counter-attack situations, which may increase their maximal speed performance during the match. Investigations in elite handball players showed similar results in both sexes related to the volume of displacements and actions, with higher demands for high-intensity actions in male compared to female beach handball players (high intensity impacts, collisions, and high-intensity accelerations and decelerations) [1, 40].

In the same way, these results were also confirmed in semiprofessional handball players in Costa Rica [6]. In this tournament, male players obtained higher values than female players in total distance, average speed, maximum speed, and total impacts. Therefore, although the variables that explain the performance in beach handball in women and men are similar, a difference in workload demands between sexes was found in the greater intensity of the actions performed by men.

To the best of the authors’ knowledge, only three previous studies have analyzed the principal components related to external demands. These studies stated that explained variance is a combination of high-intensity actions, speed changes, jumps, and impacts in basketball and soccer [25, 41, 42]. Therefore, although basketball, soccer, and beach handball are all invasion team sports, their structures provoke that the explained variance of external workload variables obtained by PCA analysis differs between them. Based on the foregoing, and on the principle of specificity and individuality, it is essential to understand that each sport and team could have their own PCA results [21]. The evidence collected in this study is a key point parameter for the behavior of external load in elite handball competitions and is essential to consider for the design of training programs and recovery processes in beach handball players.

### Limitations

The predominant strength of the present study is the use of a PCA approach based on the objectively and validated measurement of several internal and external variables to predict the workload of male and female elite beach handball players during matches. Nonetheless, our study has limitations that should be highlighted. No prior published studies were found which investigated elite beach handball players, making direct comparisons with other studies difficult. This clearly highlights the need for further examinations of the compositional nature of internal and external variables across several training demands and geographic locations. Moreover, assessments were performed over a tournament period, in which athlete´s performance could be affected depending on the match demands, as well as between 10-minutes sets and attack-defense team formation that cannot be analyzed due to data was facilitated by the tournament organizers. For these reasons, future research could explore the effect of sets, attack-defense team formation and effect of fatigue throughout the tournament to help the understanding of beach handball workload dynamics due to their influence in other team sports [31, 43, 44], as well as include technical-tactical indexes (e.g. throw or pass efficacy, type of goal scored, etc.) in principal component analysis to provide a global vision of performance in beach handball players [26]. Nonetheless, this could be a strong point, as all participants were high-level players.

## CONCLUSIONS

Five principal components were extracted that explain the internal and external workload profile of elite beach handball players (PC1: Distance_Expl_, Distance, Distance_4–7 km/h_, and Acc; PC2: Acc_Max_, Acc_3–4 m/s_, Dec_4–3 m/s_; PC3: Jumps_Avg_ Take-Off, Jumps_Avg_ Landing, and PL_RT_; PC4: Distance_> 18.1 km/h_ and Speed_Max_; PC5: HR_Avg_ and Step Balance). The distribution of principal components differs between sexes, while selected variables are similar. In addition, although the variables that explain performance in beach handball are similar between sexes, the higher intensity of actions performed by males must be considered for specific dose-response of internal and external workload during training process.

### Practical applications

Due to natural increases in interest in beach handball as an Olympic sport, its internal and external workload demands play an important role for coaches and sports professionals and represent an essential tool to exploit and sustain player technical and tactical qualities throughout the season. Sport scientist, athletes and coaches may understand that internal and external load in beach handball differ between sexes. Principal component analysis is a statistical technic that may help to understand global locomotion behavior in beach handball playing, identifying time-related distance, heart rate and accelerations as fundamental in beach handball physical-physiological load.
